# Motivational counselling and SMS-reminders for reduction of daily sitting time in patients with rheumatoid arthritis: a descriptive randomised controlled feasibility study

**DOI:** 10.1186/s12891-016-1266-6

**Published:** 2016-10-18

**Authors:** T. Thomsen, M. Aadahl, N. Beyer, M. L. Hetland, K. Løppenthin, J. Midtgaard, R. Christensen, B. A. Esbensen

**Affiliations:** 1 Copenhagen Center for Arthritis Research (COPECARE), Center for Rheumatology and Spine Diseases, Centre of Head and Orthopaedics, Rigshospitalet, Ndr. Ringvej 57, DK-2600 Glostrup, Denmark; 2 Research Centre for Prevention and Health, Rigshospitalet, The Capital Region of Denmark, Glostrup, Denmark; 3 Department of Public Health, Faculty of Health and Medical Sciences, University of Copenhagen, Copenhagen, Denmark; 4 Musculoskeletal Rehabilitation Research Unit, Bispebjerg and Frederiksberg Hospitals, University of Copenhagen, Copenhagen, Denmark; 5 Department of Clinical Medicine, Faculty of Health and Medical Sciences, University of Copenhagen, Copenhagen, Denmark; 6 The DANBIO registry, Center for Rheumatology and Spine Diseases, Centre for Head and Orthopaedics, Rigshospitalet, Ndr. Ringvej 57, DK-2600 Glostrup, Denmark; 7 University Hospitals Centre for Health Research, Copenhagen University Hospital, Rigshospitalet, Glostrup, Denmark; 8 Musculoskeletal Statistics Unit, The Parker Institute, Copenhagen University Hospital, Bispebjerg and Frederiksberg, Copenhagen, Denmark

**Keywords:** Individually tailored behavioural intervention, Acceptability, Text messages, Pain, Fatigue, Self-efficacy, ActivPAL, Cardiovascular biomarkers

## Abstract

**Background:**

Patients with rheumatoid arthritis (RA) spend a high proportion of their waking time in sedentary behaviour (SB) and have an increased risk of cardiovascular disease. Reduction of SB and increase in light intensity physical activity has been suggested as a means of improvement of health in patients with mobility problems. Short-term intervention studies have demonstrated that SB can be reduced by behavioural interventions in sedentary populations. To evaluate descriptively the feasibility of recruitment, randomisation, outcome assessments, retention and the acceptability of an individually tailored, theory-based behavioural intervention targeting reduction in daily sitting time in patients with RA.

**Methods:**

A randomised, controlled trial with two parallel groups. RA patients >18 years of age and Health Assessment Questionnaire (HAQ) score < 2.5 were consecutively invited and screened for daily leisure time sitting > 4 h. The 16-week intervention included 1) three individual motivational counselling sessions and 2) individual text message reminders aimed at reducing daily sitting time. The control group was encouraged to maintain their usual lifestyles. Outcomes were assessed at baseline and after the 16 week intervention. Daily sitting time was measured using an ActivPAL3^TM^ activity monitor. The study was not powered to show superiority; rather the objective was to focus on acceptability among patients and clinical health professionals.

**Results:**

In total, 107 patients were invited and screened before 20 met eligibility criteria and consented; reasons for declining study participation were mostly flares, lack of time and co-morbidities. One patient from the control group dropped out before end of intervention (due to a RA flare). Intervention participants completed all counselling sessions. All procedures regarding implementation of the trial protocol were feasible. The daily sitting time was reduced on average by 0.30 h in the intervention group unlike the control group that tended to increase it by 0.15 h after 16 weeks.

**Conclusions:**

This study shows that an individually tailored behavioural intervention targeting reduction of SB was feasible and acceptable to patients with RA.

**Trial registration:**

The Danish Data Protection Agency (ref.nb. 711-1-08 - 20 March 2011), the Ethics Committee of the Capital Region of Denmark (ref.nb. H-2-2012-112- 17 October 2012), clinicaltrials.gov (NCT01969604 - October 17 2013, retrospectively registered).

## Background

Rheumatoid arthritis (RA) is a chronic inflammatory disease affecting 0.5–1.0 % of the population in developed countries [1]. It is associated with pain and fatigue, decreased health-related quality of life and co-morbidity such as cardiovascular diseases and osteoporosis [1–4]. Short-term health benefits from exercise have been documented in patients with RA [5, 6]. However, the majority of patients with RA do not meet recommendations of moderate to vigorous physical activity (MVPA) [7] and spend a higher proportion of their waking hours sitting than the general population (71 % and 62 % respectively of waking hours, objectively measured by accelerometer) [8]. Sedentary behaviour (SB) is defined as any waking behaviour characterized by energy expenditure <1. 5 metabolic equivalents (METs) while in a sitting or reclined position [9]. Recent population-based observational studies measuring SB as self-reported TV-viewing [10–14], leisure time sitting [15, 16] and total daily sitting time [13, 15, 17] have suggested that SB may be a behaviourally independent risk factor for cardiovascular disease [10, 16, 17], premature death [10, 11, 13], cardio-metabolic biomarkers [12, 15] and certain types of cancer [14]. Accordingly, a meta-analysis of interventions aiming to reduce SB in adults has proposed beneficial effects of interventions targeting reduction of SB specifically [18]. Moreover, a review from 2012 has suggested that aiming to increase PA levels among patients with mobility disability should not focus solely on increasing MVPA, but should also target reduction of sitting time and increase of light intensity activity, the “non-exercise” part of the activity continuum [19]. This approach may prove feasible for improving and maintaining health in patients with chronic disease and mobility limitations. Nonetheless, there are no previous intervention studies specifically targeting reduction of SB in patients with RA. Intervention studies using objective measures of sitting time as outcome measures in older [20], overweight and obese adults [21, 22], and in desk-based office employees [23, 24] have demonstrated that sitting time can be reduced through behavioural interventions [20, 21], use of sit-stand workstations [23] and height-adjustable workstations, combined with face-to-face coaching and telephone support [24]. Moreover, energy expenditure can be increased by reducing TV-viewing time [22], possibly as a result of replacing sedentary TV-viewing time with activities that require higher energy expenditure e.g. standing or moving about. Means to reduce SB have included motivational counselling for encouraging people to make behavioural changes to improve their health. This has shown positive effects on health behaviours, such as alcohol- and tobacco use or sedentary behaviour in a range of medical care settings [25] and on waist circumference and insulin in healthy adults [26]. In addition, studies that have included text messages addressing changes in daily physical activity as part of the behavioural intervention have shown positive effects on physical activity levels and weight in clinical settings [27].

Whether reduction of SB is a beneficial health promotion strategy in patients with RA is yet to be determined. We wanted to investigate this by means of an individually tailored behavioural intervention in a randomized controlled trial. However, considering the novelty of this approach and the fluctuating severity of RA, we found it essential to determine first whether the methods, practicalities and demands of such a study were acceptable for patients with RA and for those involved in implementing the intervention. We wanted to evaluate individual reactions to the intervention and time and resources spent on recruitment, intervention and assessments.

Therefore, the aim of the present study was to evaluate descriptively the feasibility of recruitment, randomisation, retention, and outcome assessments at baseline and immediately after the intervention. Furthermore, to evaluate the acceptability of an individually tailored, theory-based behavioural intervention targeting reduction in daily sedentary behaviour in patients with RA.

## Methods

### Design

A 16 week randomised controlled trial with focus on feasibility and acceptability, and with blinded outcome assessors.

### Setting and participants

Participants were recruited from the rheumatology outpatient clinic at Rigshospitalet, Glostrup. Inclusion criteria were: RA (defined by the 1987 American College of Rheumatology/European League Against Rheumatism classification criteria for RA) [28]; > 18 years of age; disease duration > 1 year; a Health Assessment Questionnaire (HAQ)-score < 2.5; understanding/speaking Danish; daily leisure-time sitting > 4 h and access to a mobile phone. Exclusion criteria were: vigorous intensity physical activity > 8 h per week; HAQ-score > 2.5 and pregnancy. The project coordinator (TT) screened medical records systematically for potentially eligible patients who were consecutively invited by letter. A few days later TT conducted a telephone-based screening to ensure that the patients met the eligibility criteria regarding SB and vigorous physical activity using the Physical Activity Scale (PAS 2.1) [29]. Eligible patients were invited to an individual information session with TT. Informed consent was obtained from each patient immediately following the session or during the following two days.

### Randomisation and assessor blinding

Randomisation was conducted by an external collaborator, ZiteLab Aps (http://www.zitelab.dk/), which was not involved in the assessments or intervention. It was performed immediately after baseline measurements via computer-generated “random numbers” with randomisation to either an intervention group (*n* = 10) or a control group (*n* = 10). The outcome assessors were blinded to participants’ allocation status throughout the study.

### Behaviour change intervention

The 16 week intervention was conducted at Rigshospitalet, Glostrup, and consisted of 1) three individual motivational counselling sessions and 2) individual text message reminders targeting reduction of SB. TT monitored overall programme adherence, programme logistics and the dispatch of the text messages.

#### Training of project staff prior to the intervention

Five of the project staff (three project nurses, BAE and TT) conducted the motivational counselling sessions and attended a one-day tailored motivational interviewing (MI) training course supervised by a clinical psychologist with broad experience in MI and health behaviour change. The course featured MI theory, principles and interview techniques. During the study, the project staff received continuous supervision by the psychologist including discussion of conducted counselling sessions.

Two occupational therapists with broad clinical experience in rheumatology conducted the outcome assessments. Prior to study start the therapists received supervision from BAE and TT on how to perform the assessments. In addition, they were trained to save all recorded data through an online interface via a tablet. During the study the assessors were continuously supervised by TT in order to secure a uniform data collection methodology.

#### Structure and content of the intervention

Participants received three individual motivational counselling sessions. The first session took place on the day the participants were randomised and informed about allocation status; the second and third sessions took place two and ten weeks after the first session. The sessions were conducted either in a room at the rheumatology outpatient clinic or in the research unit, where the same interviewer (BAE, TT or a project nurse) conducted all three sessions.

**Fig. 1:**
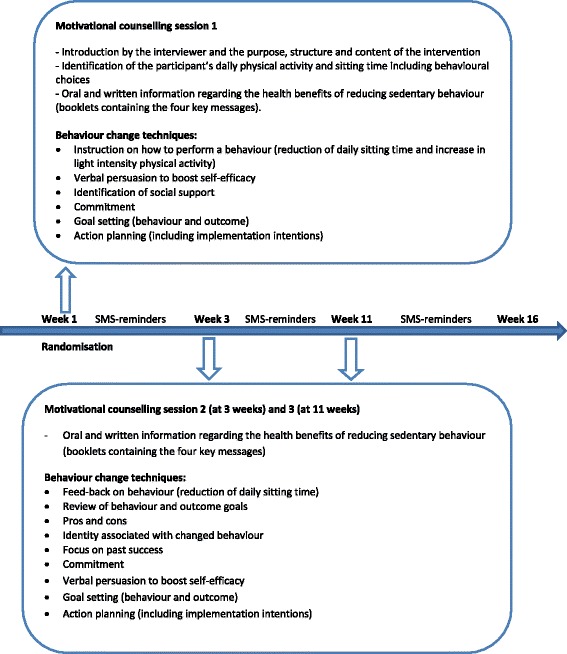
Time schedule of the 16 week intervention period including the applied behaviour change techniques according to the intervention taxonomy by Michie et al. [33]

The intervention was based on behavioural choice theory [30] which addresses how people replace the choice of an unhealthy reinforcing behaviour with less reinforcing and more healthy alternatives. Self-efficacy [31] was chosen because it in particular addresses an individual’s own belief in what one can do under different conditions with the skills the individual holds. Furthermore, motivational interviewing techniques were included in the intervention [32]. The first motivational counselling session commenced by identifying the participant’s physical activity and SB patterns during a typical weekday and the interviewer reporting the health benefits of reducing SB. The session also incorporated individual behavioural goal-setting and action-planning for change in SB with the participants identifying daily behavioural choices and describing possible behaviour alternatives (e.g. ‘*Every day at work I will hold my telephone conversations standing up’* or ‘*I will leave the remote at the TV tonight so I’ll have to get up whenever I want to change the channel’*). In session two and three, behavioural goals were reviewed, including discussion of pros and cons of the outcomes of the behaviour, identity associated with the changed behaviour, and feed-back on the behaviour from the interviewer. Potentially, behavioural goals were modified or new ones were set. During the three sessions the interviewer was supported by a number of MI sheets, including prompt sheets with relevant questions for the participants, a “decisional balance” work sheet and sheets to assess importance and self-belief in reducing daily SB. A time schedule of the intervention and the applied behaviour change techniques according to the intervention taxonomy by Michie et al. [33] is provided in Fig. 1.

#### Key messages

The intervention focused on four key messages regarding reduction of SB; 1) reduce TV-viewing, 2) substitute sitting with standing when possible – at work and/or at home, 3) break up prolonged sitting by standing up frequently and 4) maximum 30 min of sitting per episode. During the intervention the participants were provided with four booklets containing each of the key messages with specific suggestions and ideas for reduction of daily sitting time.

#### Individual text message reminders

After each motivational counselling session, based on the participants’ individual behavioural goal(s) and their gender, age, partner status, housing, work status and hobbies, an external communications consultant drafted individually tailored text messages to each one reminding them of their behavioural goal(s). In the motivational counselling session the participants had decided frequency and timing of the text messages. Participants could receive a maximum of one reminder per weekday, i.e. a maximum of five reminders per week. SMS-Track Aps, (https://www.sms-track.com/) developed and monitored the technical system supporting the sending of the text messages.

### Control group

Participants in the control group were encouraged to maintain their usual lifestyle during the 16-week intervention period. They were not in contact with the project staff until the post intervention assessment.

### Feasibility and acceptability of the intervention

TT documented all components of the recruitment and screening procedure. This included documentation of the number of invited and screened patients and reasons for exclusion or declining study participation. Adherence to the intervention was monitored using an interviewer-administered log sheet in which information on participant, behavioural goals, text message reminders and acceptability and progress of the sessions were recorded. That also included acceptability of frequency and content of text message reminders. Furthermore, by the end of the intervention period, the participants evaluated the intervention by filling in a short questionnaire about the structure and content of the intervention, potential changes in everyday habits and the impact of the intervention on physical activity patterns of friends and families.

### Outcome measures

Assessments took place at baseline prior to randomisation and at end of the 16-week intervention period. Detailed description of the measurements is reported elsewhere [34]. Outcome measures are briefly described below.

*Change in daily sitting time* was objectively measured using an ActivPAL®3TM Activity Monitor (PAL Technologies, Glasgow, UK), which has demonstrated reliability and validity for measuring posture and motion during everyday physical activities [35] and is currently considered the best choice for measurements of sitting/lying. The monitor was worn anteriorly on the upper right thigh, enfolded in waterproof dressing and kept in place by adhesive tape. The participants wore the ActivPAL for a seven-day period for each assessment. The ActivPAL does not distinguish between sitting and lying posture, which is why the participants, during the assessments, kept a diary of their sleeping time in order to separate this from their sitting/lying time while awake. Changes in daily sitting time were also measured by self-report using the Physical Activity Scale 2.1 (PAS 2.1) [29] and by specific questions about the total time spent sitting and the longest uninterrupted time spent sitting during both work and leisure [36]. *Change in number of breaks in daily sitting time* was also measured using the ActivPAL monitor. *Change in pain* was measured using the Visual Analogue Scale (VAS) which transforms the subjective experience of pain by putting a mark on a 100 mm line, ranging from “no pain” to “worst imaginable pain” [37]. *Change in fatigue* was measured using the 20-item Multidimensional Fatigue Inventory (MFI) [38]. It classifies fatigue in five dimensions; 1) general fatigue, 2) physical fatigue, 3) mental fatigue, 4) reduced activity and 5) reduced motivation. The scores range from 4 to 20, with higher scores indicating higher levels of fatigue. Fatigue was also measured by VAS [37]. *Change in functional function* was measured by the Health Assessment Questionnaire (HAQ), which contains 20 items with four possible answers in eight categories of function within daily activities. The highest scores of each dimension are summarized and divided by 8, resulting in a possible range of total scores (HAQ score) from 0 (no difficulty) to 3 (unable to do) [39]. *Change in Health-Related Quality of Life (HR-QoL)* was measured using the generic Short Form Health Survey (SF-36) [40], which was divided into two SF-36 summary scales; 1) the physical component summary scale (PCS) and 2) the mental component summary scale (MCS) and each domain is scored from 0 (low) - 100 (high). *Change in self-efficacy* was measured using the 10-item General Self-Efficacy Scale (GSES) and possible response categories are “not at all true”, “hardly true”, “moderately true” and “exactly true”, yielding a total score between 10 (low) – 40 (high) [41]. GSES measures the general sense of perceived self-efficacy in coping with a variety of demands in life. *Changes in biomarkers and blood pressure;* non-fasting venous blood samples were drawn. Total cholesterol, high-density lipoprotein cholesterol (HDL), low-density lipoprotein cholesterol (LDL) and triglycerides were measured by an enzymatic method on the Vitros 5.1 FS from Ortho Clinical Diagnostics. C-reactive protein (CRP) and HbA1c were measured on the Vitros 5.1 FS and G8 HPLC Analyzer from TOSOH. In addition, after 5–10 min of resting (sitting), blood pressure was measured digitally three times at the right upper arm and the average of the three measurements was recorded. *Changes in weight, waist circumference, BMI and waist-hip-ratio;* Weight was measured to the nearest 0.1 kg in light clothing and without shoes; Waist circumference was measured to the nearest 0.5 cm midway between the lower rib margin and the iliac crest and hip circumference was measured to the nearest 0.5 cm at the point yielding the maximum circumference over the buttocks. Subsequently, body mass index (BMI, kg/m^2^) and waist-hip ratio were calculated. At baseline each participant’s height was also measured to the nearest centimetre without shoes.

#### Descriptive variables

Additional descriptive data included self-reported data about demography, lifestyle (smoking and alcohol) and medical history (consumption of painkillers and co-morbidities). Additionally, clinical data (medical treatment, RA duration, DAS28-score, IgM Rheumatoid Factor and anti-CCP) were obtained from the DANBIO database at Rigshospitalet, Glostrup [42].

### Data management and statistical analyses

All data (except for ActivPAL-data and blood test results) were entered directly by the participants and the two assessors through an online interface via a tablet. All data were stored in unidentifiable form (using participant-numbers) in the DANBIO database [42]. The scoring of the standardised questionnaires was carried out according to the guidelines from the instrument developers. ActivPAL-data were processed using the ActivPAL software, version 7.2.32. All calculations were performed using SAS software (v. 9.3; SAS Institute Inc., Cary, NC, USA); descriptive statistics were computed for presentation of participants’ characteristics, including medians, means (M) and standard deviations (SD) for continuous data and frequencies (%) for categorical data. Changes in outcome measures from baseline to follow-up were reported as within-group differences in the intervention and the control group separately, by medians, means (M) and standard deviations (SD), and between-group differences were summarised as mean changes and standard deviations between intervention and control groups.

## Results

### Recruitment and sample characteristics

**Fig. 2:**
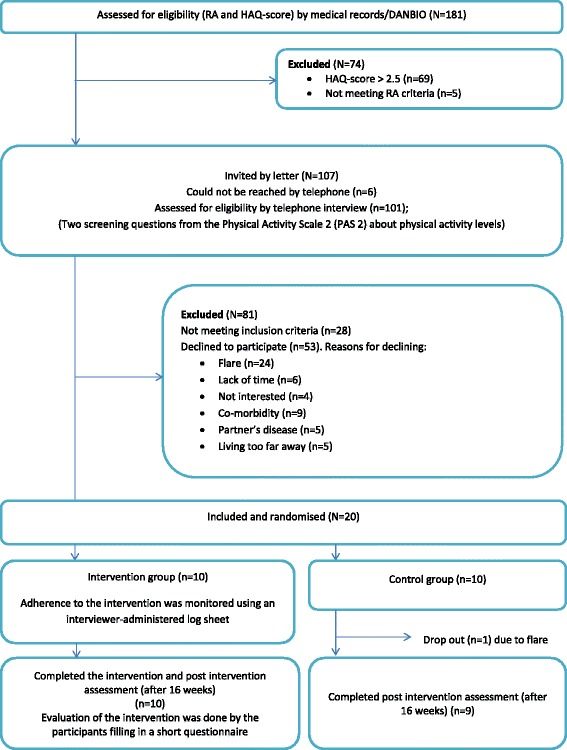
The participants’ flow through the study

The recruitment process commenced on 1^st^ of November 2012 and continued until 28^th^ of January 2013. TT screened 181 medical journals; 107 patients were potentially eligible and invited by letter before 20 were identified as meeting all eligibility criteria and had consented to participate. Reasons for declining study participation were mostly flares, i.e. a worsening in disease activity e.g. painful and tender joints, and lack of time and co-morbidities. The flow of participants through the trial is presented in Fig. 2.

There were no major differences at baseline between the intervention and the control group in terms of demographic and clinical characteristics (Table 1). All participants completed baseline assessments. One participant in the control group dropped out before end of intervention period due to disease flare. The remaining 19 participants completed post intervention assessment including all self-reported and objective assessments.

**Table 1:** Baseline characteristics of the participants by allocated group and total. Data are presented as numbers (N) unless otherwise stated

Characteristic	Intervention group (*N* = 10)	Control group (*N* = 10)	Total
Women	6	6	12 (60 %)
Age (years); M(SD)	64.5 (8.5)	54.0 (14.0)	59.3 (12.5)
Cohabiting	2	6	8 (42 %)
Highest attained education			
Primary school	5	2	7 (35 %)
High school	0	3	3 (15 %)
Short to middle higher education	4	5	9 (45 %)
Long higher education (university)	1	0	1 (5 %)
Occupation			
Unemployed	1	0	1 (5 %)
Employed full time	1	1	2 (10 %)
Employed part time	1	1	2 (10 %)
Disease-related retirement	1	3	4 (20 %)
Age-related retirement	6	5	11 (55 %)
Smoking	3	2	5 (25 %)
Drinks of alcohol per week; median (Q1,Q3)	2.0 (2.0,5.0)	2.0 (1.0,6.0)	2.0 (0.0,6.0)
RA duration (years); median (Q1,Q3)	10.0 (8,2)	4.0 (2.0,8.0)	8.0 (4.0,15.0)
Medical treatment (biologics)	6	6	12 (60 %)
DAS-28; M(SD)	3.4 (1.6)	2.8 (1.0)	3.1 (1.3)
CRP; median (Q1,Q3)	7.0 (5.0,16.0)	5.0 (5.5,9.0)	6.5 (5.0,12.5)
Comorbidity	8	9	17 (85 %)
Daily sitting time Hours/day; M(SD)	10.7 (1.9)	9.5 (1.5)	10.1 (1.8)
Breaks in sitting time N/day; M(SD)	50.0 (18.0)	48.0 (5.0)	49.0 (13.0)
Self-reported leisure sitting, Hours/day; M(SD)	4.0 (1.6)	5.6 (1.9)	4.8 (1.9)
Physical function; median (Q1,Q3)^b^	0.6 (0.4,1.0)	0.6 (0.4,1.1)	0.6 (0.4–1.1)
Fatigue; M(SD)^c^			
General fatigue	11.3 (3.5)	13.5 (2.7)	12.4 (3.3)
Physical fatigue	11.7 (2.9)	12.3 (2.7)	12.0 (2.7)
Reduced activity	11.2 (2.8)	11.5 (2.8)	11.4 (2.7)
Reduced motivation	9.0 (3.8)	10.6 (3.6)	9.8 (3.7)
Mental fatigue	10.4 (3.5)	10.5 (2.5)	10.5 (3.0)
Pain; median (Q1,Q3)^d^	20.5 (6.0,4)	28.0 (22.0,33.0)	26.5 (12.0,37.5)
HR-QoL; M(SD)^e^			
SF-36-PCS	42.7 (7.6)	37.1 (6.8)	39.6 (7.5)
SF-36-MCS	50.4 (7.6)	54.4 (7.6)	52.6 (7.7)
Self-efficacy; M(SD)^f^	30.2 (3.5)	29.2 (5.0)	29.7 (4.2)
Lipids (mmol/l)			
Cholesterol (total); M(SD)	5.7 (1.2)	5.2 (1.4)	5.4 (1.3)
HDL; M(SD)	1.5 (0.4)	1.4 (0.4)	1.5 (0.4)
LDL; M(SD)	3.2 (1.1)	3.0 (1.1)	3.2 (1.1)
Triglyceride; median (Q1,Q3)	1.7 (1.3,2.2)	1.0 (0.9,1.8)	1.4 (0.9,2.1)
HbA1c (mmol/mol)^a^; median (Q1,Q3)	5.6 (5.4,5.8)	5.6 (5.5,5.7)	5.6 (5.4,5.8)
Blood pressure (mmHg); M(SD)			
Systolic	133.8 (18.0)	122.0 (23.4)	128.2 (21.0)
Diastolic	81.0 (10.2)	75.0 (10.8)	78.1 (10.7)
Weight (kg); M(SD)	84.3 (22.0)	72.4 (10.8)	78.7 (18.2)
Waist circumference (cm); M(SD)	88.9 (24.5)	84.4 (9.3)	86.8 (18.6)
BMI; M(SD)	28.7 (6.5)	21.9 (4.2)	25.5 (6.5)
Waist-hip-ratio; M(SD)	0.9(0.2)	0.9 (0.1)	0.9 (0.1)

^a^Participants were not fasting before measurement of HbA1c

^b^Higher scores indicate higher degree of disability

^c^Higher scores indicate higher level of fatigue

^d^Higher scores indicate higher level of pain

^e^Higher scores indicate better HR-QoL

^f^Higher scores indicate higher level of self-efficacy

### Intervention adherence

All intervention participants completed the three individual motivational counselling sessions which ranged from 29 to 102 min (average 68 min). Furthermore, all participants set behavioural goals and planned actions to reduce their daily sitting time. The number of behavioural goals for each participant ranged from 1 to 5. The behavioural goals were reduction of TV-viewing, to go for an evening stroll rather than turning on the TV immediately after dinner, inviting the partner or other family members to join on these strolls, going for an extra walk with the dog, and to work standing up for an hour or two after lunch and to get up more frequently to go to the printer. All ten participants chose to receive text message reminders each week reminding them of their behavioural goals and action plans. Frequency of reminders ranged from 1 to 4 per week for each participant. Examples of behavioural goals and subsequent text message reminders according to the four key messages are provided in the Appendix.

### Participants’ response to and evaluation of the intervention

Response to and evaluation of the intervention were obtained through both a) the interviewer-administered log sheets that were collected during the individual motivational counselling session and b) the evaluation questionnaires that were distributed after the intervention (*n* = 9).

From the individual motivational counselling session we learned that the intervention in general was well accepted by the participants because of its individual approach and limited number (three) of scheduled visits to the hospital. Likewise, meeting the same interviewer throughout all three motivational counselling sessions was welcomed by the participants. The participants understood rather quickly that the main health promoting focus of the intervention was not to increase daily MVPA but a low-intensity approach of increasing light-intensity physical activity by reducing daily sedentary behaviour. All interviewers made efforts to explain this focus during the motivational counselling sessions. However, two participants still had trouble understanding this approach and thus trouble in articulating how they would reduce their sitting time, and they chose a single and more general behavioural goal to reduce sitting time. In addition, all participants reported some changes in daily habits, activities or bodily experiences during or after the intervention. This included e.g. reduced TV-viewing, more walking and more frequent interruptions of sitting time. They considered that these changes had become part of their daily life, but not necessarily easily maintained ones. Regarding text message reminders, the participants’ perception of the messages varied regarding applicability, frequency and quality The participants were satisfied with the wording and content of the messages, and most of the participants felt motivated to pursue their behavioral goals and action plans when receiving the message and they re-read them several times. However, two participants did not use the messages at all after the second counselling session.

From the evaluation questionnaire we found that participants reported physical changes in terms of e.g. reduced pain from back, hips or knees and weight reduction. Two participants reported increased pain from their hips and one experienced increased pain from the knees. They attributed this pain to the increased amount of walking. Eight participants found the frequency and duration of sessions suitable while two expressed a need for more sessions with the interviewer.

### Adverse events and completeness of data

Two participants reported redness and itching on the thigh after a couple of days wearing the ActivPAL monitor. Advice about changing the adhesive tape more frequently was given accordingly. Complaints about wearing the ActivPAL monitors did not affect the completeness of ActivPAL-data. All assessor – and participants entered data were complete. Four participants were not familiar with the use of a tablet and chose to report data via a questionnaire in paper format. These data were later entered into the DANBIO database by a project staff.

### Outcome measures

The main behavioural and clinical outcomes are presented in Table 2, which shows within-group and between-group changes from baseline to follow-up after the intervention period. The mean change in daily sitting time was −0.30 (SD 1.90) hours per day in the intervention group versus 0.15 (SD 1.43) hours per day in the control group (Table 2). The sample size was too small to draw any valid conclusions on within- and between-group changes in outcome measures. However, the aim of the present study was not to detect effects of the intervention but to study the feasibility of study procedures, and procedures in relation to collection of outcome measures proved feasible and acceptable to participants and staff.

**Table 2:** Change from baseline in assessed outcomes at end of the 16-week intervention

Variables	Intervention group (*N* = 10) Median, M(SD)	Control group (*N* = 9) Median, M(SD)	Difference between groups M(SD)
Daily sitting time Hours/day	−0.04,−0.30 (1.90)	0.18, 0.15 (1.43)	−0.46 (1.70)
Breaks in sitting time N/day	1.00, 1.00 (5.00)	−5.00, −3.00 (9.5)	5.00 (13.00)
Leisure time sitting Hours/day	0.00, 0.25 (1.72)	−0.50, −1.00 (2.45)	1.25 (2.01)
Physical function	0.00, −0.05 (0.12)	0.00, 0.07 (0.28)	−0.12(0.22)
Fatigue			
General fatigue	0.50, 0.40 (3.80)	1.00, 0.77 (2.54)	−0.38 (3.27)
Physical fatigue	−3.50, −0.90 (5.57)	1.00, 0.44 (3.00)	−1.34 (4.54)
Reduced activity	−0.50, −1.80 (4.34)	1.00, 0.88 (3.72)	−2.70 (4.06)
Reduced motivation	1.50, 1.00 (3.23)	0.00, 0.00 (2.96)	1.00 (3.10)
Mental fatigue	−1.50, −1.50 (4.43)	0.00, 0.00 (2.41)	−1.50 (3.62)
Pain mm	4.00, 3.00 (20.52)	2.00, 7.22 (18.20)	−4.22 (19.45)
HR-QoL^a^			
SF36-PCS	−7.50, −8.38 (5.50)	−20.00, −21.22 (9.47)	12.85 (7.87)
SF36-MCS	1.50, 4.38 (8.08)	−2.00, −0.55 (7.20)	4.90 (7.63)
Self-efficacy (GSES)	0.50, 0.70 (2.58)	−1.00, −1.33 (1.87)	2.03 (2.28)
Lipids (mmol/L)			
Cholesterol (total)	−0.45, −0.66 (0.78)	0.20, 0.24 (0.38)	−0.90 (0.63)
HDL	−0.02, −0.046 (0.15)	0.00, 0.07 (0.36)	−0.12 (0.27)
LDL	−0.10, 0.14 (0.89)	0.10, 0.24 (0.60)	−0.10 (0.77)
Triglycerid	−0.36, −0.43 (0.42)	0.05, 0.12 (0.32)	−0.55 (0.38)
HbA1c (mmol/mol)^b^	0.05, −0.26 (1.27)	0.30, 0.36 (0.24)	−0.62 (0.94)
Blood pressure (mmHg)			
Systolic	2.70, 4.71 (6.53)	1.60, 0.93 (9.37)	3.77 (8.00)
Diastolic	0.30, −0.27 (5.10)	−2.00, −0.45 (6.70)	0.19 (5.90)
Weight (Kg)	−0.50, 0.20 (2.14)	0.70, 0.41 (1.10)	−0.21 (1.73)
Waist circumference (cm)	0.00, 8.24 (17.38)	2.00, 5.00 (8.80)	3.25 (14.00)
BMI	−0.17, 0.07 (0.72)	0.27, 1.84 (5.03)	−1.78 (3.50)
Waist-hip-ratio	−0.01, 0.04 (0.12)	0.04, 0.04 (0.03)	0.00 (0.01)

^a^Two participants in the intervention group did not provide answers for the SF-36 questionnaire at end of intervention

^b^Participants were not fasting before measurement of HbA1c

## Discussion

The primary aim of this study was to evaluate descriptively the feasibility of recruitment, randomisation, outcome assessments, retention and the acceptability of an individually tailored, theory-based behavioural intervention targeting reduction in daily sedentary behaviour in patients with RA. The study showed that the procedures of recruitment, randomization, outcome assessments, retention and analysis were feasible and that the intervention was well accepted by the participants.

The study sample was relatively homogenous in terms of disease activity, medical treatment, duration of RA and physical function. The tailored approach, accommodating all levels of physical function, cognitive ability and disease activity, was a main feature of the intervention enabling all participants to take part fully. Adherence to the motivational counselling sessions was good and consistent with findings from a recent pilot study testing the effect of motivational counselling on medication adherence in patients with RA [43].

The participants in our study responded positively to the counselling style, especially regarding the individual approach and meeting the same interviewer throughout all sessions. In accordance with this, previous research has shown that patients with RA prefer education about the disease and its treatment and management to be delivered on a one-to-one basis by health professionals [44]. The individual approach enabled our participants to make specific and individual behavioural goals and action plans to reduce their sitting time, comprising simple initiatives such as standing up every half an hour while watching a movie or going for a stroll every evening. However, we cannot be certain whether a group-based intervention and experience sharing between the participants would have facilitated other perspectives on SB, and thus inspired different behavioural goals or approaches in reducing sitting time. Being in a group might have been beneficial for those participants who had difficulty understanding the concept of reduction of SB as an non-exercise health promotion strategy [45] and thus trouble articulating how they would reduce their daily sitting time. During the motivational counselling sessions the interviewers experienced the need to give repeated examples of how to reduce daily sitting that did not necessarily included exercise or MVPA. A consideration for future studies might be to ensure that all motivational interviewers understand how to deliver this information in the best way to the participants. In general, the content, duration and frequency of the motivational sessions appeared to be acceptable, feasible and of benefit.

To the best of our knowledge, this is the first study documenting the use of text message reminders in an intervention targeting reduction of daily SB. A previous pilot study targeting increase in daily physical activity in adults suggested that interventions applying text message reminders on behavioural goals and action plans are not more effective than those without such reminders [46]. However, the text message reminders in that study were generic, and not, as they were in our study, tailored to the individual. In our study, the receipt of the tailored text message reminders was diverse among the participants. We were attentive to let the wording in the messages be as positive as possible, reflecting an assumption that the participants were already carrying out their behavioural goals and action plans. In keeping the intervention as individualized as possible there was no minimum in the frequency of text messages. This could possibly lead to diversity in the intensity of the intervention among the participants. However, a recent systematic review found that message tailoring and personalization and varying message frequency were significantly associated with greater intervention efficacy in physical activity interventions [47]. Finally, Hall et al. (2015) argue in their systematic review that to date no strong recommendations can be drawn on what characteristics work better than others in text message-based interventions [27].

The strengths of this study included using objective measurement of daily sitting time and the two assessors’ considerable experience and expertise in working with patients with RA. In addition, the assessors were blinded to group allocation. We believe that the simple structure of the intervention with only three required visits to the hospital and the flexibility the two assessors displayed in changing assessment schedules in cases of flare was reflected in the good retention rates. Only one participant dropped out due to flare, which was expected due to the fluctuating character of the disease. Even though the participants found the duration of the sessions suitable, the longest lasting motivational counselling session lasted 102 min which must be considered very time-consuming for both research purposes and for a potential implementation of the intervention in clinical practice. When the results from the final clinical study and the cost effectiveness study are available, we will be able to assess the benefits of the intervention comparing to both time spent and patients’ and health professionals’ efforts. A few recruitment challenges need to be addressed. A total of 50 % (*n* = 53) of the invited patients declined to participate and 45 % (*n* = 24) of these reported RA-related disease activity as the main reason for doing so. Several of these patients additionally reported that the disease activity was always high during winter. We carried out the recruitment during the winter (November –January), which may have influenced the number of patients willing to participate. Another recruitment challenge was the inclusion criterion of at least four hours of daily leisure-time sitting. This was based on the results from a Danish cross-sectional study showing a median score of four hours of self-reported leisure-time sitting in patients with RA [48]. Of the 28 patients not meeting eligibility criteria after the telephone-based screening, 24 (86 %) were excluded because they reported less than four hours of daily leisure-time sitting. Most of these patients were working full- or part time. Hence, they would have less leisure time per day to spend sitting. However, they could have spent most of their work day sitting and thus, potentially, still achieve health benefits by reducing daily sitting time. Accordingly, only four of the participants in our study were working. Future studies may consider changing this inclusion criterion to cover total daily sitting time and not leisure-time sitting only and to increase the minimum criterion of hours spent sitting during a day.

This feasibility study was not intended to or powered to show a statistically significant reduction in daily sitting time in the intervention group compared to the control group. However, the selection of outcome measures appears to be appropriate and acceptable and potential health benefits of this intervention among people with RA could be safely explored at larger scale. We find it appropriate to conduct a full-scale and sufficiently powered randomised controlled trial to achieve more solid conclusions for behavioural and clinical outcomes.

## Conclusion

This randomised controlled feasibility study showed that an individually tailored behavioural intervention targeting reduction of SB was feasible and acceptable to patients with RA. We recommend the testing of a similar intervention in a randomised controlled trial powered to detect an effect on daily sitting time as the primary outcome measure.

## Appendix

**Table 3:** Examples of the participants’ goals and subsequent SMS-reminders according to the four key messages

Goal	SMS-reminders
Reduction of daily TV-viewing	• Hi X. If commercials are on why not go and get a refreshing glass of water? • Hi X. Start your laundry before you sit down to watch TV and give it a check next time there are commercials. Maybe it needs drying or folding. • Hi X. Give your body a good stretch and why not do the dishes while you wait for the commercials to finish? • Hi X. Have you been sitting for long in front of the TV today? If you take your old newspapers down to the container, then you recycle and get a mouthful of fresh air at the same time. • Hi X. If you sit down in front of the TV today, then put the remote away. Your body will be pleased for the stretch every time you switch channels.
Substitution of sitting with standing when possible – at work, at home or during transportation	• Hi X. Next time the phone rings, why not hold the conversation standing up? • Hi X. Let gravity assist in digesting your lunch; raise your table and work standing up the next hour. You might even inspire your colleagues. • Hi X. This morning, on your way to work, why not stand up in the aisle for the last two stops before you get off the train? • Hi X. Are you on your way home from work? If the sun is shining, why not get off the bus a stop earlier than usual? Remember to enjoy the weather while you walk.
Break up prolonged sitting – by standing up frequently	• Hi X. Are you doing your crosswords? For every sixth word you do, get up and get yourself a glass of water or a piece of fruit. Maybe an orange, your favourite fruit. • Hi X. Anything interesting in the newspaper today? When you finish the next article, get up and stretch your legs and maybe put another log on the fire.
Maximum 30 min of sitting per episode	• Hi X. Before you sit down at your computer this afternoon start a project that needs your attention once in a while. Maybe your laundry if you have booked the machines. • Hi X. If the weather allows it, put your sewing away and take a swing down by the lake. There might be some hungry ducks if you bring some bread. • Hi X. At tonight’s poker game you can be the one getting drinks for the others, e.g. every time cards are dealt. It would surely be appreciated. • It is difficult to really appreciate the spring in the garden from a fourth floor apartment. An activity for the coffee club today could be to go down and see the blooming plants and trees.

## Abbreviations

GSES: General self-efficacy scale
HAQ: Health assessment questionnaire
HDL: High density lipoprotein cholesterol
HR-QOL: Health-related quality of life
LDL: Low density lipoprotein cholesterol
MI: Motivational interviewing
MVPA: Moderate to vigorous physical activity
PAS 2: Physical activity scale 2
RA: Rheumatoid arthritis
SB: Sedentary behaviour
SF36-MCS: SF36- mental component scale
SF36-PCS: SF36-physical component scale
VAS: Visual analogue scale
